# Supplementary material for: Strong External Electric Fields Reduce Explosive Sensitivity: A Theoretical Investigation into the Reaction Selectivity in NH2NO2∙∙∙NH3
Source: Molecules. 2023 Mar 13;28(6):2586. doi: 10.3390/molecules28062586 (PMC10058811; doi:10.3390/molecules28062586)
Supplement: Supplementary file 1 [file molecules-28-02586-s001.zip › molecules-2247143-supplementary.pdf]

Supporting information

Strong external electric fields reduce explosive sensitivity. A theoretical investigation into the reaction selectivity in $NH_2NO_2\cdots NH_3$

Fu-de Ren, Ying-zhe Liu, Xiao-lei Wang, Li-li Qiu, Zi-hui Meng, Xiang Cheng, Yong-xiang Li

Supplementary Tables

Table S1 Barriers (E_a) and bond dissociation energies (BDE) at the MP2/6-311++G(2d,p) and CCSD(T)/6-311++G(2d,p)//MP2/6-311++G(2d,p) levels.

Table S2 The optimized geometrical parameters in the different field strengths and orientations at the MP2/6-311++G(2d,p) level.

Table S3. Transition states imaginary frequencies, Gibbs energies, reaction rate constants, Wigner tunneling corrections in the absence and presence of fields of varying strengths and directions for TS1.

Table S4 Mulliken charges and APT charges of the atoms as well as the index of imaginary vibration ($\overline{A_{H7}}$ and $\overline{A_{H5}}$) of TS1 under the $-x$-direction field at the MP2/6-311++G(2d,p) level.

Table S5 Mulliken charges and APT charges of the atoms of TS2 under the $-x$-direction field at the MP2/6-311++G(2d,p) level.

Table S6 Transition states imaginary frequencies, Gibbs energies, reaction rate constants, Wigner tunneling corrections in the absence and presence of fields of varying strengths and directions for TS2.

Figure S1 $NH_2NO_2\cdots NH_3$, in which NH_3 is only as a H-bonded donor.

Figure S2 Changes of the activation distances versus field strengths in the different field orientations for transition states at the MP2/6-311++G(2d,p) level.

Figure S3 The research scheme for the effects of external electric fields on the initiation reactions in $NH_2NO_2\cdots NH_3$.

Cartesian coordinates for the optimized geometries of reactant and transition states in the different external field strengths and orientations as well as in the absence of field.

Table S1 Barriers (E_a) and bond dissociation energies (BDE) at the MP2/6-311++G(2d,p) and CCSD(T)/6-311++G(2d,p)//MP2/6-311++G(2d,p) levels.

Field	$E_{a,TS1}$(MP2)	$E_{a,TS1}$(CCSD(T))	$E_{a,TS2}$(MP2)	$E_{a,TS2}$(CCSD(T))	BDE(MP2)	BDE(CCSD(T))
No field	68.38	77.16	176.15	190.25	257.63	239.12
z-0.010	71.29	79.26	235.4	246.18	252.05	236.75
z-0.008	75.04	81.14	219.18	237.22	253.89	227.01
z-0.006	67.02	76.55	210.22	233.12	255.72	238.46
z-0.004	69.18	79.13	190.18	208.51	256.18	238.85
z-0.002	70.22	78.27	181.23	200.17	257.54	239.07
z+0.002	71.08	79.15	176.51	191.32	258.19	241.5
z+0.004	68.99	78.53	172.18	186.57	258.42	243.64
z+0.006	72.17	79.26	170.66	185.03	259.76	245.57
z+0.008	70.02	81.18	156.03	172.31	261.22	247.16
z+0.010	74.18	85.62	158.94	177.22	262.18	248.64
y-0.010	86.55	95.17	195.62	212.31	247.92	235.32
y-0.008	81.22	94.02	194.28	213.58	249.58	238.45
y-0.006	73.17	85.18	192.1	212.12	250.61	240.42
y-0.004	70.89	82.03	187.18	208.53	253.26	238.94
y-0.002	74.25	82.96	183.25	192.54	255.2	239.13
y+0.002	73.17	83.27	181.16	190.62	259.18	242.01
y+0.004	74.22	85.18	184.23	195.18	262.25	244.72
y+0.006	75.89	87.22	190.55	209.32	265.37	246.84
y+0.008	79.03	91.05	202.58	219.18	267.85	248.89
y+0.010	87.92	98.28	214.56	228.17	269.3	259.29
x+0.010	45.89	57.03	168.51	181.55		
x+0.008			169.51	181.17		
x+0.006			170.22	182.18		
x+0.004	48.02	60.18	175.36	186.05	256.03	236.36
x+0.002	50.40	60.13	174.01	188.51	256.92	239.25
x-0.002	86.41	97.14	178.4	193.06	258.5	239.25
x-0.004	100.98	116.55	179.28	194.22	261.26	242.42
x-0.006	114.02	132.23	182.03	196.53	262.74	246.98
x-0.008	143.49	161.53	183.28	195.89	267.91	250.69
x-0.010	183.98	206.28	190.18	203.24	270.18	255.38
x-0.012	202.17	207.16	205.14	218.19		
x-0.014	239.18	257.18	212.57	230.17		
x-0.016	253.22	279.06	229.32	245.18		
x-0.018	262.17	284.18	234.18	249.55		
x-0.019	275.10	288.62	242.07	256.18		
x-0.020	291.35	296.52	257.09	272.18		

Table S2 The optimized geometrical parameters in the different field strengths and orientations at the MP2/6-311++G(2d,p) level.

Field	TS1						TS2					
	$R_{O8\cdots H7}$	$R_{H7\cdots N6}$	$R_{N6\cdots H5}$	$R_{H5\cdots N1}$	$R_{N1\text{-}H4}$	$R_{H4\cdots O3}$	$R_{O8\cdots H7}$	$R_{H7\text{-}N6}$	$R_{N6\cdots H5}$	$R_{H5\text{-}N1}$	$R_{N1\cdots H4}$	$R_{H4\cdots O3}$
Nofield	1.607	1.073	1.100	1.580	1.019	2.260	2.700	1.018	1.905	1.040	1.291	1.320
z-0.010	1.483	1.128	1.137	1.523	1.018	2.247	2.244	1.020	2.129	1.030	1.288	1.314
z-0.008	1.528	1.113	1.131	1.530	1.019	2.250	2.262	1.020	2.078	1.032	1.286	1.318
z-0.006	1.553	1.099	1.123	1.545	1.017	2.255	2.307	1.020	2.033	1.033	1.285	1.320
z-0.004	1.572	1.085	1.115	1.562	1.019	2.256	2.376	1.019	1.987	1.035	1.283	1.324
z-0.002	1.591	1.077	1.108	1.573	1.019	2.258	2.491	1.018	1.945	1.038	1.287	1.322
z+0.002	1.613	1.072	1.096	1.582	1.019	2.263	3.200	1.017	1.872	1.044	1.294	1.316
z+0.004	1.627	1.067	1.088	1.588	1.018	2.265	4.019	1.016	1.872	1.045	1.298	1.310
z+0.006	1.650	1.060	1.080	1.596	1.020	2.266	3.805	1.016	1.842	1.050	1.300	1.310
z+0.008	1.658	1.057	1.075	1.600	1.020	2.269	3.948	1.016	1.827	1.054	1.304	1.307
z+0.01	1.688	1.055	1.061	1.612	1.019	2.272	4.534	1.017	1.829	1.055	1.312	1.300
y-0.010	1.681	1.060	1.072	1.618	1.021	2.245	2.735	1.016	1.907	1.043	1.297	1.315
y-0.008	1.667	1.066	1.081	1.613	1.019	2.247	2.722	1.016	1.909	1.042	1.294	1.318
y-0.006	1.640	1.068	1.090	1.600	1.019	2.252	2.713	1.016	1.908	1.042	1.293	1.318
y-0.004	1.622	1.072	1.095	1.587	1.020	2.256	2.708	1.018	1.908	1.041	1.293	1.319
y-0.002	1.610	1.072	1.097	1.583	1.019	2.260	2.703	1.018	1.906	1.040	1.292	1.320
y+0.002	1.608	1.070	1.098	1.581	1.019	2.257	2.711	1.017	1.906	1.040	1.292	1.320
y+0.004	1.626	1.068	1.092	1.592	1.018	2.256	2.713	1.018	1.907	1.041	1.293	1.318
y+0.006	1.640	1.063	1.083	1.598	1.019	2.254	2.719	1.019	1.909	1.041	1.294	1.317
y+0.008	1.658	1.061	1.072	1.610	1.020	2.254	2.725	1.017	1.912	1.043	1.295	1.315
y+0.010	1.672	1.058	1.068	1.616	1.020	2.253	2.730	1.017	1.915	1.044	1.297	1.314
x+0.010	1.715	1.052	1.071	1.635	1.018	2.221	2.218	1.016	2.075	1.025	1.307	1.326
x+0.008	1.712	1.055	1.080	1.629	1.017	2.223	2.239	1.018	2.068	1.030	1.300	1.327
x+0.006	1.714	1.052	1.083	1.625	1.018	2.236	2.327	1.017	2.005	1.033	1.299	1.323
x+0.004	1.716	1.053	1.092	1.613	1.018	2.244	2.458	1.017	1.988	1.035	1.295	1.323
x+0.002	1.658	1.062	1.095	1.598	1.018	2.252	2.534	1.018	1.943	1.037	1.293	1.322
x-0.002	1.562	1.084	1.106	1.559	1.019	2.268	3.017	1.018	1.874	1.044	1.290	1.317
x-0.004	1.521	1.097	1.114	1.534	1.019	2.278	3.314	1.018	1.853	1.047	1.289	1.313
x-0.006	1.481	1.111	1.124	1.507	1.020	2.288	2.940	1.018	1.833	1.053	1.286	1.313
x-0.008	1.443	1.127	1.137	1.476	1.020	2.300	2.956	1.019	1.809	1.058	1.284	1.311
x-0.010	1.467	1.117	1.100	1.606	1.026	2.303	2.955	1.019	1.785	1.064	1.283	1.310
x-0.012	1.563	1.089	1.076	1.727	1.028	2.300	2.956	1.020	1.756	1.072	1.282	1.308
x-0.014	1.402	1.102	1.058	1.652	1.029	2.322	2.949	1.020	1.725	1.081	1.281	1.306
x-0.016	1.331	1.145	1.128	1.418	1.027	2.350	2.932	1.021	1.687	1.092	1.281	1.304
x-0.018	1.256	1.258	1.253	1.382	1.025	2.368	2.904	1.021	1.637	1.110	1.282	1.302
x-0.019	1.620	1.283	1.280	1.300	1.025	2.371	2.882	1.022	1.600	1.125	1.283	1.302
x-0.020	1.187	1.315	1.315	1.255	1.024	2.382	2.835	1.022	1.535	1.154	1.286	1.299

Table S3. Transition states imaginary frequencies (Imν, cm^{-1}), Gibbs energies (ΔG, kJ/mol), reaction rate constants (k, s^{-1}), Wigner tunneling corrections (κ) in the absence and presence of fields of varying strengths and directions for the intermolecular hydrogen exchange path (TS1) at the MP2/6-311++G(2d,p) level

**Field**	**Imν**	**$\Delta G_{298.15}$**	**$k_{298.15\ K}$**	**$k_{298.15\ K}$**	**$\Delta G_{688\ K}$**	**$k_{688\ K}$**	**$k_{688\ K}$**
No field	368.4	70.58	2.86×10^{0}	1.132	88.62	2.68×10^{6}	1.025
z-0.010	915.8	73.52	8.73×10^{-1}	1.815	92.55	1.35×10^{6}	1.153
z-0.008	872.7	77.39	1.83×10^{-1}	1.740	93.10	1.22×10^{6}	1.139
z-0.006	492.0	68.13	7.68×10^{0}	1.235	85.62	4.53×10^{6}	1.044
z-0.004	379.3	71.55	1.93×10^{0}	1.140	84.18	5.82×10^{6}	1.026
z-0.002	378.1	72.08	1.56×10^{0}	1.139	86.03	4.21×10^{6}	1.026
z+0.002	355.6	73.02	1.07×10^{0}	1.123	88.92	2.54×10^{6}	1.023
z+0.004	320.8	71.83	1.73×10^{0}	1.100	90.41	1.96×10^{6}	1.019
z+0.006	337.0	74.03	7.11×10^{-1}	1.110	92.50	1.36×10^{6}	1.021
z+0.008	293.5	72.59	1.27×10^{0}	1.084	87.37	3.33×10^{6}	1.016
z+0.010	271.9	76.27	2.88×10^{-1}	1.072	95.52	8.02×10^{5}	1.013
y-0.010	350.6	86.55	4.56×10^{-3}	1.119	110.38	5.97×10^{4}	1.022
y-0.008	327.9	83.18	1.77×10^{-2}	1.104	106.26	1.23×10^{5}	1.020
y-0.006	342.8	76.18	2.99×10^{-1}	1.114	95.01	8.76×10^{5}	1.021
y-0.004	360.7	73.18	1.00×10^{0}	1.126	89.17	2.43×10^{6}	1.024
y-0.002	367.6	76.92	2.22×10^{-1}	1.131	92.35	1.40×10^{6}	1.025
y+0.002	335.1	75.00	4.81×10^{-1}	1.109	93.25	1.19×10^{6}	1.020
y+0.004	320.7	76.24	2.92×10^{-1}	1.100	95.18	8.51×10^{5}	1.019
y+0.006	303.5	78.24	1.30×10^{-1}	1.090	91.00	1.77×10^{6}	1.017
y+0.008	349.2	82.66	2.19×10^{-2}	1.118	106.36	1.20×10^{5}	1.022
y+0.010	341.8	90.03	1.12×10^{-3}	1.114	108.27	8.63×10^{4}	1.021
x+0.010	262.0	48.04	2.54×10^{4}	1.067	62.18	2.72×10^{8}	1.013
x+0.004	260.0	50.17	1.08×10^{4}	1.066	60.54	3.63×10^{8}	1.012
x+0.002	310.4	52.99	3.45×10^{3}	1.094	62.15	2.74×10^{8}	1.018
x-0.002	445.0	88.37	2.19×10^{-3}	1.192	112.26	4.30×10^{4}	1.036
x-0.004	547.3	103.71	4.49×10^{-6}	1.291	128.71	2.42×10^{3}	1.055
x-0.006	689.6	115.53	3.82×10^{-8}	1.462	138.53	4.35×10^{2}	1.087
x-0.008	868.6	143.57	4.67×10^{-13}	1.733	180.02	3.08×10^{-1}	1.138
x-0.010	649.2	183.67	4.41×10^{-20}	1.410	227.18	8.08×10^{-5}	1.077
x-0.012	420.2	199.01	9.05×10^{-23}	1.172	248.26	2.03×10^{-6}	1.032
x-0.014	1124.3	236.28	2.68×10^{-29}	2.228	288.33	1.84×10^{-9}	1.231
x-0.016	1157.9	246.19	2.91×10^{-31}	2.303	336.05	4.38×10^{-13}	1.245
x-0.018	1528.7	255.11	1.34×10^{-32}	3.271	340.29	2.09×10^{-13}	1.426
x-0.019	1633.0	263.62	4.34×10^{-34}	3.591	365.03	2.76×10^{-15}	1.487
x-0.020	1638.5	278.33	1.15×10^{-36}	3.609	368.81	1.43×10^{-15}	1.490

Table S4 Mulliken charges and APT charges (in bold) of the atoms as well as the index of imaginary vibration ($\overline{A_{\mathrm{H7}}}$ and $\overline{A_{\mathrm{H5}}}$) of TS1 under the −*x*-direction field at the MP2/6-311++G(2d,p) level.

	**0.000**	**-0.002**	**-0.004**	**-0.006**	**-0.008**	**-0.010**	**-0.012**	**-0.014**	**-0.016**	**-0.018**	**-0.019**	**-0.020**
O8	-0.444	-0.448	-0.452	-0.455	-0.457	-0.463	-0.474	-0.460	-0.452	-0.443	-0.439	-0.430
	**-0.901**	**-0.916**	**-0.928**	**-0.938**	**-0.946**	**-0.936**	**-0.933**	**-0.928**	**-0.927**	**-0.923**	**-0.920**	**-0.918**
H7	0.495	0.512	0.528	0.545	0.561	0.525	0.474	0.468	0.499	0.613	0.630	0.634
	**0.523**	**0.549**	**0.573**	**0.596**	**0.618**	**0.574**	**0.511**	**0.503**	**0.540**	**0.625**	**0.667**	**0.679**
N6	-0.786	-0.813	-0.840	-0.872	-0.907	-0.818	-0.718	-0.922	-1.023	-1.078	-1.145	-1.195
	**-0.600**	**-0.628**	**-0.657**	**-0.689**	**-0.721**	**-0.646**	**-0.543**	**-0.629**	**-0.638**	**-0.754**	**-0.913**	**-0.922**
H5	0.546	0.551	0.558	0.566	0.576	0.494	0.447	0.498	0.516	0.606	0.617	0.620
	**0.589**	**0.601**	**0.615**	**0.632**	**0.651**	**0.571**	**0.497**	**0.575**	**0.577**	**0.682**	**0.753**	**0.764**
N1	-0.656	-0.655	-0.654	-0.655	-0.656	-0.645	-0.693	-0.683	-0.652	-0.654	-0.612	-0.610
	**-0.849**	**-0.846**	**-0.844**	**-0.843**	**-0.843**	**-0.768**	**-0.746**	**-0.775**	**-0.783**	**-0.825**	**-0.799**	**-0.838**
$\overline{A_{\mathrm{H7}}}$	0.352	0.356	0.358	0.365	0.375	0.395	0.364	0.360	0.352	0.367	0.368	0.371
$\overline{A_{\mathrm{H5}}}$	0.355	0.376	0.399	0.412	0.421	0.371	0.357	0.353	0.340	0.421	0.430	0.433

Table S5 Mulliken charges and APT charges (in bold) of the atoms of TS2 under the $-x$-direction field at the MP2/6-311++G(2d,p) level.

	**0.000**	**-0.002**	**-0.004**	**-0.006**	**-0.008**	**-0.010**	**-0.012**	**-0.014**	**-0.016**	**-0.018**	**-0.019**	**-0.020**
O3	-0.306	-0.321	-0.340	-0.341	-0.351	-0.361	-0.371	-0.381	-0.391	-0.401	-0.406	-0.409
	**-0.751**	**-0.771**	**-0.790**	**-0.798**	**-0.813**	**-0.828**	**-0.844**	**-0.861**	**-0.880**	**-0.901**	**-0.914**	**-0.930**
H4	0.425	0.421	0.418	0.411	0.405	0.400	0.394	0.388	0.382	0.375	0.371	0.365
	**0.477**	**0.479**	**0.480**	**0.482**	**0.484**	**0.485**	**0.487**	**0.489**	**0.491**	**0.493**	**0.494**	**0.495**
N1	-0.527	-0.576	-0.620	-0.565	-0.570	-0.574	-0.579	-0.584	-0.590	-0.599	-0.606	-0.619
	**-0.796**	**-0.821**	**-0.838**	**-0.837**	**-0.848**	**-0.860**	**-0.874**	**-0.887**	**-0.903**	**-0.923**	**-0.935**	**-0.953**

Table S6. Imaginary frequencies (Imν, cm^{-1}), Gibbs energies (ΔG, kJ/mol), reaction rate constants (k, s^{-1}) and corrected reaction rate constants ($k_{298.15\ K,C}$ and $k_{688\ K,C}$, s^{-1}), Wigner tunneling corrections (κ) in the absence and presence of fields of varying strengths and directions for the 1,3-intramolecular hydrogen transference path (TS2) at the MP2/6-311++G(2d,p) level.

Field	Imν	$\Delta G_{298.15}$	$k_{298.15\ K}$	$\kappa_{298.15\ K}$	$\Delta G_{688\ K}$	$k_{688\ K}$	$\kappa_{688\ K}$
No field	1955.0	158.93	9.51×10^{-16}	4.714	179.59	3.32×10^{-1}	1.697
z-0.010	1984.7	222.84	6.05×10^{-27}	4.828	252.80	9.17×10^{-7}	1.719
z-0.008	1977.6	201.03	4.01×10^{-23}	4.800	221.13	2.33×10^{-4}	1.714
z-0.006	1970.0	188.51	6.26×10^{-21}	4.771	210.02	1.63×10^{-3}	1.708
z-0.004	1960.6	172.80	3.54×10^{-18}	4.735	198.26	1.27×10^{-2}	1.701
z-0.002	1958.1	160.25	5.59×10^{-16}	4.726	182.08	2.15×10^{-1}	1.700
z+0.002	1949.4	157.28	1.85×10^{-15}	4.693	176.73	5.47×10^{-1}	1.693
z+0.004	1947.6	153.19	9.64×10^{-15}	4.686	174.10	8.66×10^{-1}	1.692
z+0.006	1939.8	152.83	1.11×10^{-14}	4.656	170.70	1.57×10^{0}	1.687
z+0.008	1934.2	146.05	1.72×10^{-13}	4.635	169.04	2.10×10^{0}	1.683
z+0.010	1927.1	142.64	6.79×10^{-13}	4.609	163.18	5.84×10^{0}	1.678
y-0.010	1972.0	177.92	4.48×10^{-19}	4.779	202.05	6.54×10^{-3}	1.710
y-0.008	1970.3	173.51	2.66×10^{-18}	4.772	195.07	2.22×10^{-2}	1.708
y-0.006	1962.9	170.22	1.00×10^{-17}	4.744	194.35	2.51×10^{-2}	1.703
y-0.004	1958.3	166.57	4.36×10^{-17}	4.726	186.22	1.04×10^{-1}	1.700
y-0.002	1957.2	161.39	3.53×10^{-16}	4.722	183.37	1.71×10^{-1}	1.699
y+0.002	1958.9	160.26	5.56×10^{-16}	4.729	180.09	3.04×10^{-1}	1.700
y+0.004	1959.8	163.55	1.48×10^{-16}	4.732	186.81	9.39×10^{-2}	1.701
y+0.006	1960.2	172.89	3.41×10^{-18}	4.734	196.37	1.77×10^{-2}	1.701
y+0.008	1969.4	180.62	1.51×10^{-19}	4.769	205.10	3.84×10^{-3}	1.708
y+0.010	1974.0	193.18	9.51×10^{-22}	4.786	223.29	1.59×10^{-4}	1.711
x+0.010	1985.2	149.23	4.76×10^{-14}	4.829	165.63	3.81×10^{0}	1.719
x+0.008	1979.4	154.44	5.82×10^{-15}	4.807	173.52	9.59×10^{-1}	1.715
x+0.006	1973.6	154.92	4.8×10^{-15}	4.785	176.06	6.15×10^{-1}	1.711
x+0.004	1968.1	156.39	2.65×10^{-15}	4.764	177.72	4.60×10^{-1}	1.707
x+0.002	1961.5	157.08	2.01×10^{-15}	4.739	178.50	4.01×10^{-1}	1.702
x-0.002	1947.4	160.77	4.53×10^{-16}	4.685	180.67	2.75×10^{-1}	1.692
x-0.004	1939.5	162.53	2.23×10^{-16}	4.655	184.66	1.37×10^{-1}	1.686
x-0.006	1927.1	167.62	2.86×10^{-17}	4.609	188.41	7.10×10^{-2}	1.678
x-0.008	1917.0	169.78	1.20×10^{-17}	4.571	193.85	2.74×10^{-2}	1.671
x-0.010	1906.4	173.2	3.01×10^{-18}	4.531	196.72	1.66×10^{-2}	1.663
x-0.012	1895.5	183.20	5.33×10^{-20}	4.491	210.02	1.63×10^{-3}	1.656
x-0.014	1884.1	190.13	3.25×10^{-21}	4.449	216.85	4.92×10^{-4}	1.648
x-0.016	1871.8	206.82	3.88×10^{-24}	4.404	239.71	9.05×10^{-6}	1.639
x-0.018	1858.7	212.95	3.27×10^{-25}	4.357	241.63	6.46×10^{-6}	1.630
x-0.019	1851.0	220.76	2.48×10^{-28}	4.329	246.46	2.78×10^{-6}	1.625
x-0.020	1843.8	233.57	7.98×10^{-29}	4.303	248.93	1.80×10^{-6}	1.620

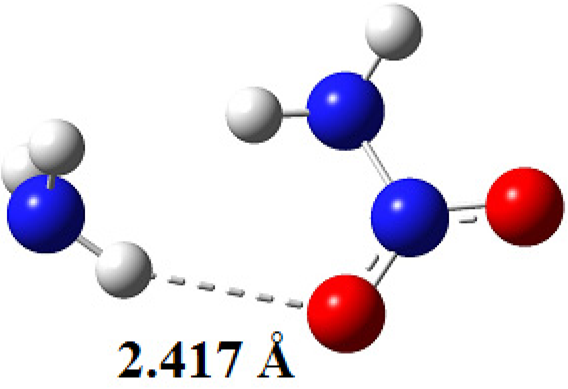

Figure S1 $NH_2NO_2 \cdots NH_3$, in which NH_3 is only as a H-bonded donor and the binding energy is 18.50 kJ/mol at the MP2/6-311++G(2d,p) level, 32.0 kJ/mol weaker than that of the $NH_2NO_2 \cdots NH_3$ in which NH_3 is not only as a H-bonded donor, but also as a H-bonded acceptor.

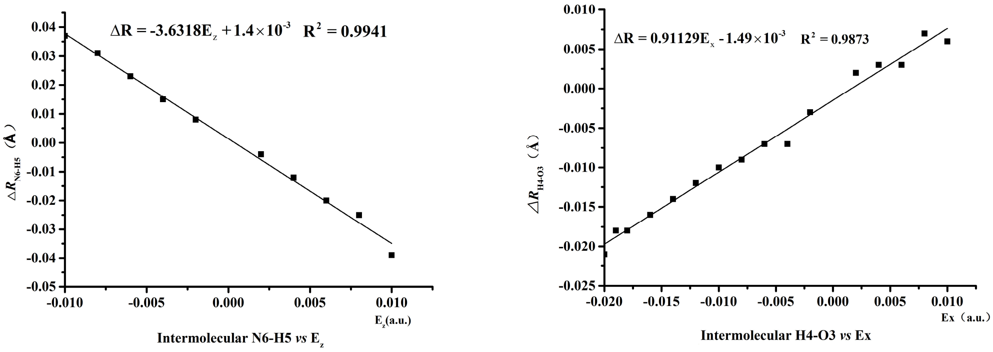

N6···H5 distances *vs*. E_Z (TS1) H4···O3 distances *vs*. E_X (TS2)

Figure S2 Changes of the activation distances versus field strengths in the different field orientations for transition states at the MP2/6-311++G(2d,p) level.

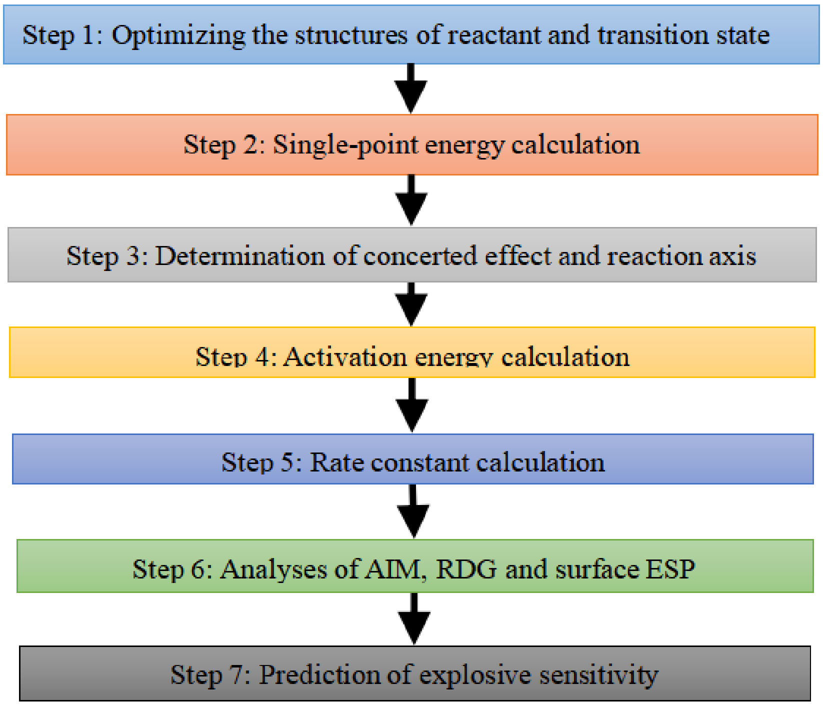

Figure S3 The research scheme for the effects of external electric fields on the initiation reactions in $NH_2NO_2{\cdots}NH_3$

Cartesian coordinates for the optimized geometries of reactant and transition states

TS1:
No field
Reactant
N,0,-0.1388307297,0.3519007669,-0.0298998831
N,0,-0.158585254,0.0031223882,1.3038966994
O,0,-1.236844475,-0.3528205867,1.7772334304
O,0,0.900358496,0.131879902,1.9225315384
N,0,2.7033834144,-0.043980718,-0.6781055687
H,0,2.889801177,0.0147217715,0.3203031404
H,0,3.1127304258,-0.9110755331,-1.0118374402
H,0,3.2094764427,0.7148728084,-1.1243657356
H,0,0.8028090931,0.1957712203,-0.4242245001
H,0,-0.9164652703,-0.0993088494,-0.497773551

Transition state

N	-0.010478778648	-0.005312276003	-0.003318686965
N	-0.009170612835	-0.000152113714	1.320231600914
H	0.970948482051	0.001744848153	-0.276105580404
O	-1.153543040165	-0.007109522133	1.910594308023
O	1.039014554030	0.010487964201	1.982432904788
N	-2.559188570973	-0.025389807317	-0.247643245452
H	-1.498157123278	-0.017793132824	-0.535555588187
H	-2.311692572416	-0.019179830342	0.796074235024
H	-3.049754405547	-0.867900889765	-0.529332097233
H	-3.064770932932	0.805591759230	-0.536769852666

X-20:
Z-Matrix found in file.
N
N,1,B1
H,1,B2,2,A1
O,2,B3,1,A2,3,D1,0
O,2,B4,1,A3,3,D2,0
N,1,B5,2,A4,3,D3,0
H,6,B6,1,A5,2,D4,0
H,6,B7,1,A6,2,D5,0
H,6,B8,1,A7,2,D6,0
H,6,B9,1,A8,2,D7,0
Variables:
B1=1.32476608

B2=1.01823686
B3=1.2819809
B4=1.24345158
B5=2.57792323
B6=1.09523203
B7=1.06236915
B8=1.0153425
B9=1.01538045
A1=105.32706857
A2=117.60384661
A3=121.86083285
A4=95.57267284
A5=20.40458392
A6=72.76626011
A7=120.8995876
A8=121.12028983
D1=-179.94544462
D2=0.05589426
D3=179.42228644
D4=-180.04147478
D5=0.32024234
D6=-105.85366849
D7=106.43835099

X+20:
Z-Matrix found in file.
N
N,1,B1
H,1,B2,2,A1
O,2,B3,1,A2,3,D1,0
O,2,B4,1,A3,3,D2,0
N,1,B5,2,A4,3,D3,0
H,6,B6,1,A5,2,D4,0
H,6,B7,1,A6,2,D5,0
H,6,B8,1,A7,2,D6,0
H,6,B9,1,A8,2,D7,0
Variables:
B1=1.3221599
B2=1.01898847
B3=1.2936114
B4=1.23607688
B5=2.54459155
B6=1.1057071
B7=1.08425361

B8=1.01419313
B9=1.01416269
A1=105.69919206
A2=116.86802539
A3=122.81084474
A4=95.52378695
A5=20.60154275
A6=69.88630919
A7=120.99180704
A8=120.96648871
D1=-179.67917093
D2=0.36745183
D3=178.75000348
D4=-179.39346888
D5=0.54816095
D6=-105.09118685
D7=106.1306122

X-40:
Z-Matrix found in file.
N
N,1,B1
H,1,B2,2,A1
O,2,B3,1,A2,3,D1,0
O,2,B4,1,A3,3,D2,0
N,1,B5,2,A4,3,D3,0
H,6,B6,1,A5,2,D4,0
H,6,B7,1,A6,2,D5,0
H,6,B8,1,A7,2,D6,0
H,6,B9,1,A8,2,D7,0
Variables:
B1=1.32089265
B2=1.01935414
B3=1.29927005
B4=1.23263617
B5=2.52922666
B6=1.11358831
B7=1.09657716
B8=1.01370263
B9=1.01364829
A1=105.93254452
A2=116.47458241
A3=123.28022002
A4=95.59213186

A5=20.29844094
A6=68.87702401
A7=120.89700016
A8=120.86513912
D1=-179.5199595
D2=0.53052315
D3=178.09645162
D4=-179.07037065
D5=0.85913947
D6=-104.7639165
D7=106.43050888

X+40:
Z-Matrix found in file.
N
N,1,B1
H,1,B2,2,A1
O,2,B3,1,A2,3,D1,0
O,2,B4,1,A3,3,D2,0
N,1,B5,2,A4,3,D3,0
H,6,B6,1,A5,2,D4,0
H,6,B7,1,A6,2,D5,0
H,6,B8,1,A7,2,D6,0
H,6,B9,1,A8,2,D7,0
Variables:
B1=1.3258996
B2=1.01786562
B3=1.27627887
B4=1.24732872
B5=2.59723396
B6=1.0919707
B7=1.05302189
B8=1.01601254
B9=1.01603557
A1=105.21214011
A2=117.93193144
A3=121.40328805
A4=95.65202037
A5=19.85830996
A6=74.72220198
A7=120.90878928
A8=120.87399876
D1=-180.08621575
D2=-0.0936819

D3=180.003156
D4=-180.01608943
D5=0.01057999
D6=-106.73814733
D7=106.70604175

X-60
Z-Matrix found in file.
N
N,1,B1
H,1,B2,2,A1
O,2,B3,1,A2,3,D1,0
O,2,B4,1,A3,3,D2,0
N,1,B5,2,A4,3,D3,0
H,6,B6,1,A5,2,D4,0
H,6,B7,1,A6,2,D5,0
H,6,B8,1,A7,2,D6,0
H,6,B9,1,A8,2,D7,0
Variables:
B1=1.31938513
B2=1.01973854
B3=1.30524575
B4=1.22912827
B5=2.5159466
B6=1.12434135
B7=1.11077024
B8=1.01325224
B9=1.01315745
A1=106.26740374
A2=116.08502387
A3=123.74684628
A4=95.64737975
A5=19.76314592
A6=67.92330165
A7=121.14673759
A8=120.28866178
D1=-179.21097559
D2=0.92390672
D3=177.34449269
D4=-177.73673267
D5=1.22891572
D6=-104.42319731
D7=107.01625147

X-80

Z-Matrix found in file.

```
N
N,1,B1
H,1,B2,2,A1
O,2,B3,1,A2,3,D1,0
O,2,B4,1,A3,3,D2,0
N,1,B5,2,A4,3,D3,0
H,6,B6,1,A5,2,D4,0
H,6,B7,1,A6,2,D5,0
H,6,B8,1,A7,2,D6,0
H,6,B9,1,A8,2,D7,0
      Variables:
B1=1.31787918
B2=1.02008827
B3=1.31100843
B4=1.22582452
B5=2.50283161
B6=1.13740938
B7=1.12683476
B8=1.01280635
B9=1.01285548
A1=106.61767273
A2=115.65535255
A3=124.24520474
A4=95.79106852
A5=19.10421865
A6=67.11703471
A7=120.82884635
A8=120.23606372
D1=-179.70982094
D2=0.35900574
D3=178.83263439
D4=-178.93777366
D5=0.65123997
D6=-105.30066451
D7=106.66529611
```

X-100

Z-Matrix found in file.

```
N
N,1,B1
H,1,B2,2,A1
O,2,B3,1,A2,3,D1,0
```

O,2,B4,1,A3,3,D2,0
N,1,B5,2,A4,3,D3,0
H,6,B6,1,A5,2,D4,0
H,6,B7,1,A6,2,D5,0
H,6,B8,1,A7,2,D6,0
H,6,B9,1,A8,2,D7,0

Variables:

B1=1.32423099
B2=1.02560161
B3=1.31459974
B4=1.22902223
B5=2.57678925
B6=1.09953595
B7=1.11658817
B8=1.01563818
B9=1.01180116
A1=104.33472374
A2=114.83924737
A3=124.90113353
A4=85.71997612
A5=21.56375557
A6=68.17713432
A7=114.85309395
A8=126.2302801
D1=-162.2961559
D2=18.03809513
D3=120.7864902
D4=-168.79860112
D5=25.286796
D6=-81.75266927
D7=131.1747262

X-120

Z-Matrix found in file.

N
N,1,B1
H,1,B2,2,A1
O,2,B3,1,A2,3,D1,0
O,2,B4,1,A3,3,D2,0
N,1,B5,2,A4,3,D3,0
H,6,B6,1,A5,2,D4,0
H,6,B7,1,A6,2,D5,0
H,6,B8,1,A7,2,D6,0
H,6,B9,1,A8,2,D7,0

Variables:
B1=1.32471097
B2=1.02681613
B3=1.31521818
B4=1.23054739
B5=2.60322289
B6=1.08854952
B7=1.10674758
B8=1.01684948
B9=1.01189274
A1=104.02423173
A2=114.71770185
A3=124.98728868
A4=82.56572963
A5=22.87282952
A6=69.01421569
A7=112.36354585
A8=128.92967158
D1=-160.03266011
D2=19.6423121
D3=112.08846437
D4=-171.06441625
D5=28.28533239
D6=-79.05739406
D7=134.30757294

X-160
Z-Matrix found in file.
N
N,1,B1
H,1,B2,2,A1
O,2,B3,1,A2,3,D1,0
O,2,B4,1,A3,3,D2,0
N,1,B5,2,A4,3,D3,0
H,6,B6,1,A5,2,D4,0
H,6,B7,1,A6,2,D5,0
H,6,B8,1,A7,2,D6,0
H,6,B9,1,A8,2,D7,0
Variables:
B1=1.32495867
B2=1.02771923
B3=1.31247411
B4=1.23422498
B5=2.64288296

B6=1.0758513
B7=1.08877286
B8=1.01852699
B9=1.01241144
A1=103.72248542
A2=114.86630281
A3=124.81460031
A4=78.24164226
A5=24.77403931
A6=70.75212768
A7=109.04771801
A8=132.52878978
D1=-158.54563483
D2=19.80758798
D3=102.55917696
D4=-174.59209227
D5=31.12648066
D6=-76.57005184
D7=137.61589147

X-180
Z-Matrix found in file.
N
N,1,B1
H,1,B2,2,A1
O,2,B3,1,A2,3,D1,0
O,2,B4,1,A3,3,D2,0
N,1,B5,2,A4,3,D3,0
H,6,B6,1,A5,2,D4,0
H,6,B7,1,A6,2,D5,0
H,6,B8,1,A7,2,D6,0
H,6,B9,1,A8,2,D7,0
Variables:
B1=1.32505944
B2=1.02775956
B3=1.31261747
B4=1.23413274
B5=2.64461625
B6=1.07609464
B7=1.0888029
B8=1.01854396
B9=1.01240596
A1=103.73933701
A2=114.8790224

A3=124.82818125
A4=78.17485935
A5=24.75185836
A6=70.71383816
A7=109.19900298
A8=132.35091181
D1=-158.46467261
D2=19.88301627
D3=102.42187811
D4=-174.29941285
D5=31.15988758
D6=-76.49966314
D7=137.6929242

X-200
Z-Matrix found in file.
N
N,1,B1
H,1,B2,2,A1
O,2,B3,1,A2,3,D1,0
O,2,B4,1,A3,3,D2,0
N,1,B5,2,A4,3,D3,0
H,6,B6,1,A5,2,D4,0
H,6,B7,1,A6,2,D5,0
H,6,B8,1,A7,2,D6,0
H,6,B9,1,A8,2,D7,0
Variables:
B1=1.31006812
B2=1.02403127
B3=1.34787581
B4=1.20599937
B5=2.49894421
B6=1.31480426
B7=1.31473005
B8=1.01431839
B9=1.01327905
A1=109.18197208
A2=113.06744557
A3=127.08729458
A4=95.47029446
A5=13.1371007
A6=61.58049745
A7=117.44063716
A8=117.20337167

D1=-170.04493971
D2=12.01358154
D3=154.28672267
D4=-167.78245348
D5=11.20558146
D6=-99.09675308
D7=125.67011322

TS2:
No field
Transition state
N,0,0.179619372,-0.2283193224,0.142838143
N,0,-0.1247602028,-0.0616459716,1.4036723497
H,0,1.1816847343,-0.072717186,-0.0897998946
H,0,-1.0945383133,-0.4300438482,0.1910942552
O,0,0.5823763775,0.2237800273,2.3578368918
O,0,-1.4289611332,-0.2749630091,1.4584875998
N,0,3.0302499376,0.3217192624,0.1487372851
H,0,2.9837125782,0.4858585426,1.1518750921
H,0,3.7056675296,-0.4211926919,-0.0032235565
H,0,3.4105191947,1.1618770505,-0.2763537668

X-20

N	-0.175000568660	-0.016541795048	-0.121303108722
N	0.118752837647	0.001967870491	1.152654841187
H	0.642271936788	-0.011473379305	-0.770851623221
H	-1.321324592105	-0.033711303255	0.469256368044
O	1.198679568799	0.028431022991	1.717580278770
O	-1.061889977312	-0.014329044743	1.759945333480
N	2.335441970332	0.006860291151	-1.573143079178
H	2.933584109159	0.027279189126	-0.750232437086
H	2.577704090172	0.819960715100	-2.132099782825
H	2.605879623949	-0.810835563972	-2.111996795678

X+20

N	-0.255417503917	0.042739967437	-0.167989131395
N	0.176742864421	-0.014328874366	1.064797046536
H	0.488112377599	0.040892699953	-0.891040128648
H	-1.333103169588	0.046235067744	0.545935763050
O	1.318559784159	-0.063037212947	1.502183572173
O	-0.918017689665	-0.009060554660	1.799656970689
N	2.317266873102	-0.001699898996	-1.543543059010
H	2.719343594982	-0.042756161795	-0.609710042162

H	2.699924338864	0.816281238173	-2.007177665067
H	2.640687530018	-0.817658268400	-2.053303326327

X-40

N	-0.122022267767	-0.020532320635	-0.086900440473
N	0.083296327694	0.016158623094	1.203500206988
H	0.731457311225	-0.015076627970	-0.693805844236
H	-1.304766338867	-0.044391510199	0.423764545842
O	1.116477564479	0.061560196818	1.844886861494
O	-1.142738582089	-0.006879899737	1.726499633064
N	2.351479162371	-0.001217268330	-1.592903285662
H	3.045646098424	0.021910698423	-0.849157024277
H	2.534116292342	0.807431852079	-2.180732145009
H	2.561153429749	-0.821355743163	-2.155342517961

X+40

Z-Matrix found in file.

N
N,1,B1
H,1,B2,2,A1
O,2,B3,1,A2,3,D1,0
O,2,B4,1,A3,3,D2,0
H,1,B5,2,A4,3,D3,0
N,3,B6,1,A5,2,D4,0
H,7,B7,3,A6,1,D5,0
H,7,B8,3,A7,1,D6,0
H,7,B9,3,A8,1,D7,0

Variables:

B1=1.30559576
B2=1.05970935
B3=1.34645671
B4=1.21423105
B5=1.28419035
B6=1.80300255
B7=1.01884704
B8=1.01736068
B9=1.01735739
A1=114.23302934
A2=103.59176692
A3=131.71451908
A4=75.90737622
A5=165.78795319
A6=103.47048306
A7=117.33624652

A8=117.41143768
D1=179.79853466
D2=-0.20340921
D3=-179.7534042
D4=0.00883097
D5=-0.140054
D6=115.90407972
D7=-116.22961861

X-60
Z-Matrix found in file.
N
N,1,B1
H,1,B2,2,A1
O,2,B3,1,A2,3,D1,0
O,2,B4,1,A3,3,D2,0
H,1,B5,2,A4,3,D3,0
N,3,B6,1,A5,2,D4,0
H,7,B7,3,A6,1,D5,0
H,7,B8,3,A7,1,D6,0
H,7,B9,3,A8,1,D7,0
Variables:
B1=1.30625597
B2=1.05306498
B3=1.33909053
B4=1.21611493
B5=1.28591507
B6=1.8326124
B7=1.01833102
B8=1.01682101
B9=1.0168253
A1=114.55621031
A2=103.82419931
A3=131.32256192
A4=75.87202225
A5=165.21260664
A6=102.21086602
A7=117.53111488
A8=117.41928478
D1=-179.98514901
D2=0.01887718
D3=179.98380928
D4=-0.17476666
D5=0.35087429

D6=116.08789225
D7=-115.32594283

X+60
Z-Matrix found in file.
N
N,1,B1
H,1,B2,2,A1
O,2,B3,1,A2,3,D1,0
O,2,B4,1,A3,3,D2,0
H,1,B5,2,A4,3,D3,0
N,3,B6,1,A5,2,D4,0
H,7,B7,3,A6,1,D5,0
H,7,B8,3,A7,1,D6,0
H,7,B9,3,A8,1,D7,0
Variables:
B1=1.30548354
B2=1.06120408
B3=1.3479788
B4=1.21388523
B5=1.28385962
B6=1.79660799
B7=1.01896273
B8=1.01746844
B9=1.01746556
A1=114.15224772
A2=103.55722912
A3=131.79040741
A4=75.92309412
A5=165.7903996
A6=103.60503243
A7=117.39774638
A8=117.40051628
D1=179.94909198
D2=-0.05906613
D3=-179.92627008
D4=-0.01669875
D5=-0.32728055
D6=115.75523073
D7=-116.4232273

X-80
Z-Matrix found in file.

N
N,1,B1
H,1,B2,2,A1
O,2,B3,1,A2,3,D1,0
O,2,B4,1,A3,3,D2,0
H,1,B5,2,A4,3,D3,0
N,3,B6,1,A5,2,D4,0
H,7,B7,3,A6,1,D5,0
H,7,B8,3,A7,1,D6,0
H,7,B9,3,A8,1,D7,0
Variables:
B1=1.30573489
B2=1.05824174
B3=1.34493421
B4=1.21459516
B5=1.28448568
B6=1.8094018
B7=1.01873248
B8=1.0172448
B9=1.01724227
A1=114.310453
A2=103.63966949
A3=131.6351336
A4=75.89995459
A5=165.72386336
A6=103.24318991
A7=117.32333092
A8=117.44251153
D1=-180.17634172
D2=-0.183666
D3=180.21814725
D4=0.12632091
D5=0.04355426
D6=116.02051746
D7=-115.99137451

X+80

N	-0.321191439387	0.035105800774	-0.179647815685
N	0.210446049726	0.000075255121	1.013364615625
H	0.348286944664	0.032712550088	-0.962083738885
H	-1.343983341803	0.037679388696	0.621818494634
O	1.391855703116	-0.030606036140	1.355988437460
O	-0.807916108811	0.004216828622	1.834863880928
N	2.332382715280	-0.010805752166	-1.543866771648

H	2.561782593778	-0.047136768567	-0.553193558658
H	2.768797006282	0.809462124290	-1.949266387850
H	2.713638875744	-0.833095383510	-1.998167161857

X-100

Z-Matrix found in file.

N
N,1,B1
H,1,B2,2,A1
O,2,B3,1,A2,3,D1,0
O,2,B4,1,A3,3,D2,0
H,1,B5,2,A4,3,D3,0
N,3,B6,1,A5,2,D4,0
H,7,B7,3,A6,1,D5,0
H,7,B8,3,A7,1,D6,0
H,7,B9,3,A8,1,D7,0

Variables:

B1=1.30518078
B2=1.06424691
B3=1.35113368
B4=1.21316037
B5=1.28332523
B6=1.7845543
B7=1.01918585
B8=1.01768789
B9=1.01768189
A1=113.99319547
A2=103.45882901
A3=131.95199757
A4=75.9298606
A5=165.93499663
A6=104.0182312
A7=117.39123898
A8=117.37810914
D1=180.66822294
D2=0.70625861
D3=-180.8391107
D4=-0.01771556
D5=-0.51607076
D6=115.68148959
D7=-116.71631899

Z-20

Z-Matrix found in file.

N
N,1,B1
H,1,B2,2,A1
H,1,B3,2,A2,3,D1,0
O,2,B4,1,A3,3,D2,0
O,2,B5,1,A4,3,D3,0
N,3,B6,1,A5,2,D4,0
H,7,B7,3,A6,1,D5,0
H,7,B8,3,A7,1,D6,0
H,7,B9,3,A8,1,D7,0
Variables:
B1=1.31058914
B2=1.03750833
B3=1.28739055
B4=1.22043767
B5=1.31973644
B6=1.94538703
B7=1.01838452
B8=1.01548309
B9=1.01547366
A1=114.5941322
A2=75.81857942
A3=130.38073806
A4=104.31837988
A5=154.73664805
A6=92.59798611
A7=120.29430214
A8=120.22051497
D1=-177.53232972
D2=-2.09297614
D3=177.99130625
D4=0.56447459
D5=0.86664301
D6=-110.57849399
D7=112.32642335

Z+20

N	-0.149241279946	0.009769008914	-0.101583063578
N	0.097332431603	0.001134163506	1.179861893573
H	0.687101144080	0.005019878130	-0.727018748631
H	-1.320388746495	0.017527262338	0.448015325364
O	1.157000900478	-0.011592578290	1.786865391900
O	-1.103551212668	0.009218526666	1.745671403799
N	2.353607364501	-0.006258596161	-1.578849552159

H	3.022297859288	-0.018899419974	-0.813191049218
H	2.563591191509	0.811477170368	-2.143968858896
H	2.546349348534	-0.819787413654	-2.155992738459

Z-40
Z-Matrix found in file.

```
 N
 N,1,B1
 H,1,B2,2,A1
 H,1,B3,2,A2,3,D1,0
 O,2,B4,1,A3,3,D2,0
 O,2,B5,1,A4,3,D3,0
 N,3,B6,1,A5,2,D4,0
 H,7,B7,3,A6,1,D5,0
 H,7,B8,3,A7,1,D6,0
 H,7,B9,3,A8,1,D7,0
       Variables:
 B1=1.31353042
 B2=1.03489212
 B3=1.28346986
 B4=1.21918705
 B5=1.31705149
 B6=1.9872198
 B7=1.01908536
 B8=1.01560669
 B9=1.01556251
 A1=114.52352175
 A2=75.89306653
 A3=130.35013654
 A4=104.21020309
 A5=151.87748123
 A6=89.69249097
 A7=121.21773301
 A8=121.8419305
 D1=-178.20296006
 D2=-1.52565847
 D3=178.55238694
 D4=0.24620764
 D5=0.86686434
 D6=-108.59278541
 D7=110.48843602
```

Z+40
Z-Matrix found in file.

N
N,1,B1
H,1,B2,2,A1
H,1,B3,2,A2,3,D1,0
O,2,B4,1,A3,3,D2,0
O,2,B5,1,A4,3,D3,0
N,3,B6,1,A5,2,D4,0
H,7,B7,3,A6,1,D5,0
H,7,B8,3,A7,1,D6,0
H,7,B9,3,A8,1,D7,0
Variables:
B1=1.30265162
B2=1.0454716
B3=1.29834937
B4=1.22187094
B5=1.33023601
B6=1.87154798
B7=1.0160031
B8=1.01664784
B9=1.01664338
A1=118.64370866
A2=75.67506797
A3=130.98455221
A4=104.30211979
A5=171.83961663
A6=114.35831228
A7=111.21190537
A8=111.21528024
D1=-180.02754857
D2=0.0206345
D3=180.01884165
D4=179.93051573
D5=-180.24923403
D6=-301.0866818
D7=-59.40868389

Z-60
Z-Matrix found in file.
N
N,1,B1
H,1,B2,2,A1
H,1,B3,2,A2,3,D1,0
O,2,B4,1,A3,3,D2,0
O,2,B5,1,A4,3,D3,0

N,3,B6,1,A5,2,D4,0
H,7,B7,3,A6,1,D5,0
H,7,B8,3,A7,1,D6,0
H,7,B9,3,A8,1,D7,0
Variables:
B1=1.31790388
B2=1.03315189
B3=1.28508402
B4=1.21766766
B5=1.31488821
B6=2.03268641
B7=1.01962409
B8=1.01593682
B9=1.01563334
A1=113.88746263
A2=75.57243027
A3=130.33394629
A4=104.13179202
A5=149.95475099
A6=87.37517589
A7=120.87904661
A8=124.27506664
D1=-166.16326877
D2=-11.79085212
D3=168.79556408
D4=1.73121132
D5=6.3648589
D6=-101.0983475
D7=114.54740621

Z+60

N	-0.039682674490	-0.001398480124	-0.035611880527
N	0.030428490692	-0.000614414119	1.263048788730
H	0.862310461283	-0.000708510490	-0.573890833217
H	-1.280593379862	-0.002298685985	0.352640537847
O	0.992058984461	0.000744613667	2.018931084239
O	-1.242325761227	-0.001568005009	1.661652544055
N	2.395697647373	0.000607704113	-1.594733738195
H	3.249677596146	0.005042111248	-1.044039514378
H	2.440655762786	0.811710916033	-2.206570290995
H	2.445871872129	-0.813909246083	-2.201616700524

Z-80
Z-Matrix found in file.

N
N,1,B1
H,1,B2,2,A1
H,1,B3,2,A2,3,D1,0
O,2,B4,1,A3,3,D2,0
O,2,B5,1,A4,3,D3,0
N,3,B6,1,A5,2,D4,0
H,7,B7,3,A6,1,D5,0
H,7,B8,3,A7,1,D6,0
H,7,B9,3,A8,1,D7,0
Variables:
B1=1.32239305
B2=1.0315668
B3=1.28608256
B4=1.21612544
B5=1.31274174
B6=2.07820065
B7=1.02008936
B8=1.01640997
B9=1.01572895
A1=113.32692005
A2=75.32012988
A3=130.27717453
A4=104.06855727
A5=148.38221158
A6=85.27997368
A7=118.93559512
A8=128.11283802
D1=-161.27150648
D2=-16.07758394
D3=164.76947839
D4=0.87255942
D5=12.39371315
D6=-93.0260448
D7=119.32363154

Z+80

N	-0.007375234783	-0.001653929614	-0.019021990300
N	0.019786052556	-0.001662286246	1.279221327468
H	0.909253343992	-0.000878308414	-0.538759402561
H	-1.263639883283	0.002056657991	0.329983612864
O	0.953840522432	-0.004432582044	2.071129038268
O	-1.267544453050	0.001328559542	1.637047298085
N	2.400411313912	0.000793624077	-1.594389837859

H	3.292948832800	0.004340846515	-1.108692787618
H	2.406475218138	0.810217642390	-2.210968626235
H	2.409943287208	-0.812502220810	-2.205738632504

Z-100
Z-Matrix found in file.

N
N,1,B1
H,1,B2,2,A1
H,1,B3,2,A2,3,D1,0
O,2,B4,1,A3,3,D2,0
O,2,B5,1,A4,3,D3,0
N,3,B6,1,A5,2,D4,0
H,7,B7,3,A6,1,D5,0
H,7,B8,3,A7,1,D6,0
H,7,B9,3,A8,1,D7,0

Variables:

B1=1.32740138
B2=1.03012506
B3=1.28824699
B4=1.21440953
B5=1.31078732
B6=2.12881078
B7=1.01587507
B8=1.0203087
B9=1.01707661
A1=112.67279231
A2=74.98409685
A3=130.20429766
A4=104.0016595
A5=146.79150785
A6=133.84095908
A7=83.76848425
A8=114.73593505
D1=-156.73105242
D2=-20.12034391
D3=160.99319081
D4=-1.07369322
D5=127.56106659
D6=21.35990221
D7=277.5527353

Z+100
Z-Matrix found in file.

N
N,1,B1
H,1,B2,2,A1
O,2,B3,1,A2,3,D1,0
O,2,B4,1,A3,3,D2,0
H,1,B5,2,A4,3,D3,0
N,3,B6,1,A5,2,D4,0
H,7,B7,3,A6,1,D5,0
H,7,B8,3,A7,1,D6,0
H,7,B9,3,A8,1,D7,0
Variables:
B1=1.29625438
B2=1.05537491
B3=1.226162
B4=1.33811297
B5=1.31154641
B6=1.82910595
B7=1.01772662
B8=1.01681931
B9=1.01905235
A1=121.71827073
A2=131.67708891
A3=104.44123806
A4=75.42348455
A5=160.73357463
A6=113.33090382
A7=120.09910528
A8=105.62066171
D1=-0.37363752
D2=179.63092786
D3=-179.56097731
D4=-179.68805356
D5=88.41356942
D6=214.90117145
D7=-26.25129388

0 field Reactant:
N,0,0.1315512978,-0.3477164016,0.1549957505
N,0,0.2024178136,-0.0114519864,1.4903546951
H,0,0.8868602495,0.1133007097,-0.339183404
O,0,-0.8300012992,-0.153636111,2.149555932
O,0,1.2963527893,0.3475744522,1.9236516742
N,0,-2.7380492168,0.0234710704,-0.3742172461
H,0,-0.8261590755,-0.1944349912,-0.1998490218

H,0,-2.8822720489,-0.0442098805,0.6305718656
H,0,-3.2501053109,-0.7405830812,-0.8044715352
H,0,-3.173727979,0.8861289397,-0.6852553203

X-Reactant

N	0	-0.217235	0.371967	0.171327
N	0	-0.485107	-0.011050	1.466995
H	0	0.784115	0.225251	-0.050710
O	0	0.437530	0.094559	2.275816
O	0	-1.635687	-0.373764	1.720416
N	0	2.667004	-0.011395	-0.151015
H	0	2.850771	-0.007609	0.849457
H	0	3.187807	0.764555	-0.549517
H	0	3.078275	-0.861652	-0.524699
H	0	-0.893111	-0.065585	-0.443826

X+20-Reactant

N	0	-0.266916	0.339103	0.104534
N	0	-0.409452	-0.010957	1.431489
H	0	0.705060	0.187355	-0.199051
O	0	0.588181	0.124560	2.146446
O	0	-1.522726	-0.372401	1.802500
N	0	2.667228	-0.006668	-0.136463
H	0	2.612023	0.064220	0.877113
H	0	3.229234	0.770672	-0.468252
H	0	3.169403	-0.860094	-0.358585
H	0	-0.997673	-0.110513	-0.435488

X+80-Reactant

N	0	-0.209689	0.287806	-0.022178
N	0	-0.263905	0.064188	1.340375
H	0	0.685809	-0.022190	-0.401867
O	0	0.816766	0.104685	1.947001
O	0	-1.366270	-0.092037	1.842377
N	0	2.845270	-0.209657	-0.210282
H	0	2.515270	-0.085843	0.745959
H	0	3.397859	0.603734	-0.458357
H	0	3.449903	-1.023004	-0.235636
H	0	-1.051467	-0.074482	-0.458663

X-80-Reactant

N	0	-0.022465	0.341511	0.307398
N	0	-0.694528	0.036916	1.458254
H	0	0.997394	0.119490	0.336957

O	0	-1.909114	-0.198581	1.353432
O	0	-0.061973	0.076761	2.510597
N	0	2.806724	-0.228754	0.042758
H	0	3.321312	0.022633	0.884074
H	0	3.067641	-1.190445	-0.161502
H	0	3.211465	0.332006	-0.703757
H	0	-0.540553	-0.021045	-0.482950

X-160-Reactant

N	0	0.286229	-0.261650	1.100046
N	0	-0.972256	0.068280	0.813334
O	0	-1.848504	-0.152747	1.680679
O	0	-1.217058	0.538289	-0.307208
N	0	2.647016	0.495464	-0.133366
H	0	3.096367	1.299025	0.304177
H	0	3.306193	-0.271574	0.002121
H	0	2.671533	0.687990	-1.132771
H	0	1.107353	0.100445	0.509988
H	0	0.405491	-0.500253	2.076016

X-200-Reactant

N	0	0.086284	0.098858	1.048053
N	0	-1.219853	0.120614	0.906236
O	0	-1.994046	-0.060796	1.913317
O	0	-1.719556	0.323145	-0.238144
N	0	2.813398	0.319773	-0.150773
H	0	3.034573	1.260157	-0.488062
H	0	3.461776	0.103587	0.613834
H	0	2.995565	-0.342834	-0.908663
H	0	1.810955	0.259841	0.169202
H	0	0.213268	-0.079078	2.048014
